# Amelioration of contusion-induced muscle injury via regulation of the NLRP3 inflammasome

**DOI:** 10.7150/ijms.119730

**Published:** 2026-01-01

**Authors:** Yen-Peng Lee, Chien-Chao Chiu, Liang-Ju Chiu, Yi-Hsun Chen, Wen-Ching Huang

**Affiliations:** 1 National Research Institute of Chinese Medicine, Ministry of Health and Welfare, Taipei 112, Taiwan.; 2 Department of Exercise and Health Science, National Taipei University of Nursing and Health Sciences, Taipei 112, Taiwan.; 3 National Laboratory Animal Center, National Applied Research Laboratories, Taipei 115, Taiwan.; 4 Graduate Institute of Sports Science, National Taiwan Sport University, Taoyuan 333, Taiwan.

**Keywords:** inflammasome, cytokine, muscle contusion, inflammation, sport injury

## Abstract

Inflammasomes, including NLRP3 (nucleotide-binding oligomerization domain, leucine-rich repeat, and pyrin domain-containing protein 3), participate in regulating immune activation and inflammation. Nonetheless, their specific roles in muscle contusion, one of the most common forms of sports injury, remain unknown. To address this, we investigated the role of NLRP3 in muscle contusion in *Nlrp3*-knockout (KO) mice, using a model of mass-drop injury (MDI)-induced contusion of the gastrocnemius muscle. Wild-type (WT) and *Nlrp3*-KO mice were assigned to four experimental groups: (1) WT-N: wild-type without MDI (control); (2) NLRP3-N: *Nlrp3*-KO without MDI; (3) WT-I: WT with MDI; and (4) NLRP3-I: *Nlrp3*-KO with MDI. Following MDI, tissue and serum samples were collected at 0, 48, 96, and 192 h. Muscle injury, recovery, and the extent of inflammation were evaluated by measuring body and muscle weight, serum biochemical markers, complete blood counts, and via histopathological immunohistochemical analysis. In the early phase following contusion, muscle weight was lower in the NLRP3-I group than in the WT-I group; however, in the later stages, it was significantly greater in the NLRP3-I group. Serum aspartate aminotransferase, lactate dehydrogenase, and creatine kinase were significantly lower in the NLRP3-I group than in the WT-I group, indicating reduced muscle damage following *Nlrp3* knockout. The white blood cell and neutrophil counts were markedly higher in the WT-I group than in the NLRP3-I group. Based on histopathology, the NLRP3-I group exhibited less severe muscle injury, reduced tumor necrosis factor (TNF)-α, interleukin (IL)-6, CD68, CD206 and Caspase-3 expression, and reduced fibrosis, indicating a diminished contusion-induced inflammatory response and improved regeneration relative to the WT-I group. *Nlrp3* knockout thus ameliorated contusion-induced muscle injury and inflammation. We hypothesize that NLRP3 regulates TNF-α and IL-6, with *Nlrp3* downregulation reducing contusion-related muscle damage and fibrosis. NLRP3 thus represents a promising therapeutic target for treating sports injuries and for rehabilitation.

## Introduction

Contusions and strains account for approximately 60-70% of all sports-related injuries, with muscle contusions comprising one-third of all sports injuries. These soft-tissue injuries, most often affecting the quadriceps and gastrocnemius muscle, typically result from blunt force trauma, such as a kick, fall, or blow, and are common in contact sports as well as from accidental contact in non-contact sports. Among soccer players, rotator cuff contusions represent nearly half of all shoulder injuries, with a minority of cases progressing to more severe injuries, such as rotator cuff tears 1. In a survey on the prevalence of exercise-related injuries at fitness centers 2, exercise duration and class type were associated with different types of injuries; while muscle strains and bruises were the most common injuries, muscle cramps, spasms, and contusions (including hematomas and bruises) were more frequent in classes shorter than an hour. In the different trial, contusions were most common in the knee, followed by the shoulder 3.

The immediate symptoms of contusion typically include pain, swelling, and discoloration. Injuries to skeletal muscle not only damage muscle cells but may also lead to capillary rupture, infiltrative bleeding, inflammation, oxidative stress, and fibrosis, with the severity depending on the extent of the injury 4. Within the complex inflammatory response to contusion, neutrophils and macrophages may exert different functions at different stages of pathogenesis and recovery. A standardized non-invasive contusion injury model has been proposed as being ideal for investigating immune responses to mechanical injury of skeletal muscle 5. Tumor necrosis factor (TNF)-α, interleukin (IL)-6, and free radicals play important roles in repairing muscle damage 6, 7. In the inflammatory phase, neutrophils and macrophages exhibit chemotactic movement toward the injured lesions for phagocytosis. M1 macrophages, especially in the early stages, participate in the inflammatory response and contribute to damage. M2 macrophages, in contrast, release various growth factors that directly accelerate myocyte replication and regeneration 8. M2 macrophages also stimulate regulatory T cells to secrete cytokines such as IL-4 and IL-10 to activate and differentiate satellite cells, thereby improving the capacity for muscle regeneration 9.

Pattern-recognition receptors (PRRs), expressed on immune cells, are categorized as toll-like receptors (TLRs) when expressed on the cell membrane and as nucleotide-binding and oligomerization domain-like receptors (NLRs) when expressed in the cytoplasm. NLRs function as inflammasomes, with NLRPs (nucleotide-binding oligomerization domain, leucine-rich repeat, and pyrin domain-containing proteins) representing the largest NLR family. Four distinct inflammasomes (NLRP1, NLRP3, NLRC4, and AIM2), associated with caspase activation and pro-inflammatory cytokine secretion, have been identified 10. Inflammasomes play a pivotal role in the host's innate defense system, and inflammasome dysregulation has been reported in several inflammatory, immune, and metabolic disorders 11. The NLRP3 inflammasome, occurring primarily in monocytes and macrophages, participates in cardiomyocyte injury and immune disorders, based on animal model studies 12-14. In inclusion body myositis, *Nlrp3* and its associated genes related to inflammasome assembly, T-cell migration, and MHC-I expression are highly co-upregulated 15. In *Nlrp3*-knockout (KO) mice with sepsis, serum interleukin-1β (IL-1β) was reduced, mitigating muscular atrophy; the same authors proposed that inhibiting IL-1β activation might prevent inflammation-induced muscle failure 16.

Nonetheless, the precise role of NLRP3 in skeletal muscle injury, including the specific cell types and cytokines involved in modulating damage severity and regeneration following contusion, remains insufficiently understood. We hypothesized that downregulating NLRP3 will attenuate muscle injury and promote regeneration, potentially by modulating the key regulatory factors involved in muscle repair. To test this, we used a skeletal-muscle contusion NLRP3-KO mouse model to investigate the role of NLRP3 in inflammation and regeneration following sports-related muscle injury.

## Material and Methods

### Animals and treatments

The nine-week-old male C57BL/6Narl mice, raised under specific pathogen-free conditions, were obtained from the National Laboratory Animal Center (NLAC, Taiwan). *Nlrp3*-KO mice (B6.129S6-*Nlrp3^tm1Bhk^*/J) were purchased from the Jackson Laboratory (Bar Harbor, ME, USA). The animals were fed a standard laboratory chow diet (No. 5001, PMI Nutrition International, Brentwood, MO, USA), provided with distilled water* ad libitum*, and appropriately housed with a 12:12 h light-dark cycle at 21 ± 1 °C and 55%**-**65% humidity. After one week of acclimation, the animals were randomly divided into four groups (*n* = 24 per group for each indicator tested): (1) WT-N: wild-type without injury (control); (2) NLRP3-N: *Nlrp3*-KO without injury; (3) WT-I: WT with mass-drop injury (MDI); and (4) NLRP3-I: *Nlrp3*-KO with MDI.

### Contusion induction and sample collection time-points

The hindlimbs of the mice were subjected to MDI-induced contusion as previously described 17, with slight modifications. Briefly, a 50 g weight was dropped from a height of 60 cm onto the medial surface of the left gastrocnemius muscle after the mouse was anesthetized with 3-4% isoflurane. Six mice from each group were then sacrificed via CO_2_ asphyxiation followed by cardiac puncture, at 0, 48, 96, and 192 h after injury. Blood samples were collected into tubes with or without anticoagulant for clinical chemistry and complete blood count (CBC) analysis. Gastrocnemius muscle tissue was preserved in 10% formalin and paraffin-embedded for histopathological examination and immunohistochemical (IHC) staining.

### Clinical chemistry and CBC

Serum samples were centrifuged at 2700 ×*g* for 10 min. Muscle injury was assessed by measuring serum aspartate aminotransferase (AST), lactate dehydrogenase (LDH), and creatine kinase (CK) levels using an automatic analyzer (HITACHI 7080, Hitachi, Tokyo, Japan). The anticoagulated blood for complete CBC assay was treated with ethylenediaminetetraacetic acid. Total blood cells, differential leukocytes, erythrocytes, and platelets were quantified using the Bayer Hematology System (ADVIA 2010 Autoanalyzer, Bayer Diagnostics, Leverkusen, Germany).

### Histological examination and Masson's trichrome staining

Muscle fixation, processing, and embedding were performed as previously described 18. Transverse sections of the gastrocnemius were obtained and stained with hematoxylin and eosin for histological observation and with Masson's trichrome stain to examine fibrosis severity. Paraffin sections were subjected to deparaffinization by placing the sections in xylene for 5 min (three times), followed by immersion in ethanol (absolute) for 5 min (twice), then at 95% for 5 min, 90% for 5 min, and 80% for 5 min, before washing with water. Sections were then stained for 5 min using Weigert's hematoxylin from the Masson trichrome staining kit (HT15, Merk, Rahway, NJ, USA), then washed with water. The sections were then stained with Ponceau S solution for 10 min, washed with water, and counterstained with Aniline blue solution for 5 min. Finally, the sections were treated with 1% acetic acid for 2 min, dehydrated, mounted, and observed under a microscope.

### IHC staining

Paraffin-embedded muscle sections were deparaffinized, rehydrated, and subjected to antigen retrieval, followed by incubation with 3% H_2_O_2_ to eliminate endogenous peroxidase activity. The sections were incubated at 4 °C overnight with rabbit anti-mouse TNF-α (1:100) polyclonal antibodies (AF-410, Novus Biologicals, Centennial, CO, USA), goat anti-mouse IL-6 (1:100) polyclonal antibody (AF-406, Novus Biologicals), rabbit anti-mouse CD206 (1:500) antibody (#24595, Cell Signaling, Danvers, MA, USA), rabbit anti-mouse CD68 (1:200) antibody (ab125212, Abcam, Waltham, MA, USA), and rabbit anti-mouse cleaved Caspase-3 (1:400) antibody (#9661, Cell Signaling, Danvers, MA, USA). They were then incubated with horseradish peroxidase-conjugated anti-rabbit (87-9963, Invitrogen, Waltham, MA, USA) or anti-goat polymer (MP-7405, VectorLabs, Newark, CA, USA), and signals were detected after adding a chromogenic substrate (3-amino-9-ethylcarbazole [AEC] or 3,3′-diaminobenzidine [DAB]). The sections were counterstained with hematoxylin and mounted for histological analysis.

### Statistical analysis

Statistical analyses were performed using SPSS 22.0 (IBM, Armonk, NY, USA). Results are presented as the mean ± SD. Differences between groups were analyzed using two-way ANOVA with Fisher's protected least significant difference (LSD) for multiple comparisons. Differences were considered significant at *p* <0.05.

## Results

### Body and muscle weight

**Figure 1:**
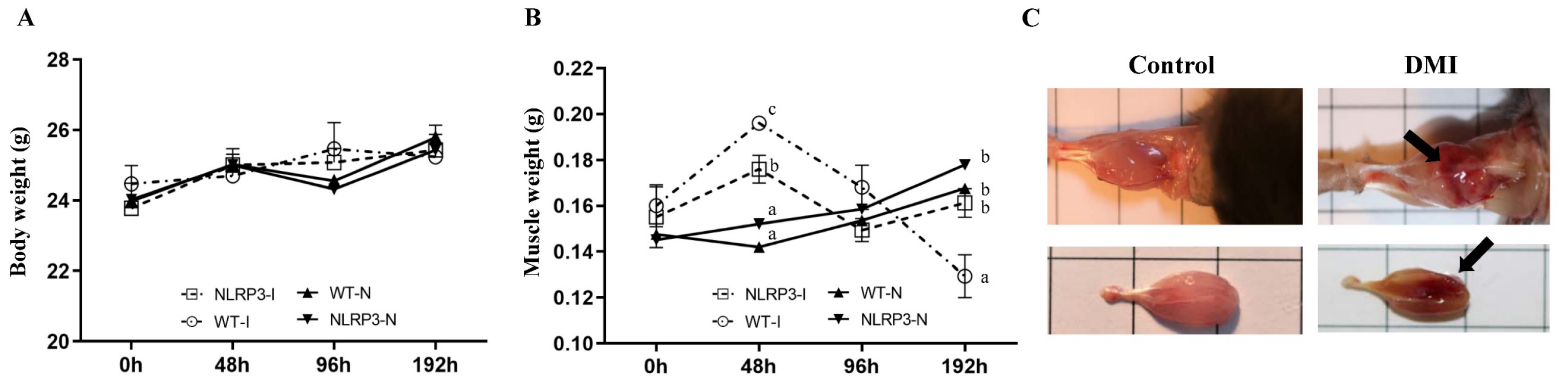
Mouse growth (A) and gastrocnemius muscle weight (B) at different time-points after mass-drop injury (MDI) in *Nlrp3*-knockout (KO) and C57BL/6Narl mice. (C) Muscle injury gross morphology. '-I', with injury; '-N', non-injured control. Different superscript lowercase letters identify groups with significant differences (*p* < 0.05). An arrow indicates the MDI-induced hemorrhage.

Body weight did not vary significantly between the WT-N, NLRP3-N, WT-I, and NLRP3-I groups at any time-point (Figure 1A). However, gastrocnemius muscle weight varied significantly among the groups at 48 and 192 h, being higher in the contusion groups (WT-I and NLRP3-I) than in the non-injury controls (WT-N and NLRP3-N). At 48 h, gastrocnemius muscle weight was significantly higher in the WT-I group than in the NLRP3-I group, and at 96 h, it was lowest (by a significant margin) in the NLRP3-I group (Figure 1B). In terms of gross structure, the gastrocnemius muscle exhibited a spindle shape, smooth surface, and normal muscle appearance, whereas the impacted surface of the injured muscle exhibited a large area of red hemorrhagic lesions (indicated by arrows) (Figure 1C).

### Clinical chemistry

**Figure 2:**
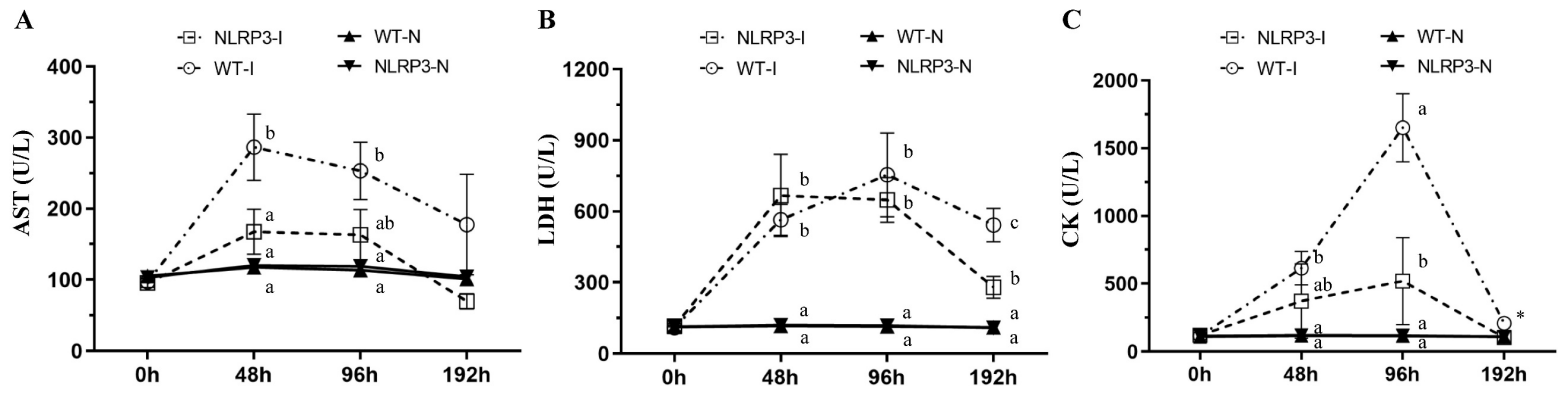
Injury-associated biochemical marker levels following mass-drop injury (MDI) in *Nlrp3*-knockout and C57BL/6Narl mice. (A) Aspartate aminotransferase (AST); (B) lactate dehydrogenase (LDH); (C) creatine kinase (CK). '-I', with injury; '-N', non-injured control. Different lowercase superscript letters identify groups with significant differences (*p* < 0.05). *, highest in the WT-I group, by a significant margin.

Muscle injury caused by MDI may be associated with several biochemical variables, including AST, LDH, and CK. At 48 and 96 h post-contusion, AST concentration was significantly higher in the WT-I group than in the NLRP3-I and non-contusion groups (WT-N and NLRP3-N) (Figure 2A); at 192 h, however, no significant differences in AST were observed among the groups. At 48 and 96 h, LDH was significantly higher in the WT-I and NLRP3-I groups than in the non-contusion groups, with no significant difference between the WT-I and NLRP3-I groups; at 192 h, it was significantly lower in the NLRP3-I group than in the WT-I group, but remained significantly higher than in the WT-N and NLRP3-N groups (Figure 2B). At 48 h, CK was significantly elevated, being significantly higher in the WT-I group than in the WT-N and NLRP3-N groups; at 96 h, it was significantly lower in the NLRP3-I group than in the WT-I group but remained significantly higher than in the WT-N and NLRP3-N groups (Figure 2C). Even at 192 h, CK was highest (by a significant margin) in the WT-I group (Figure 2C).

### Complete blood count

**Figure 3:**
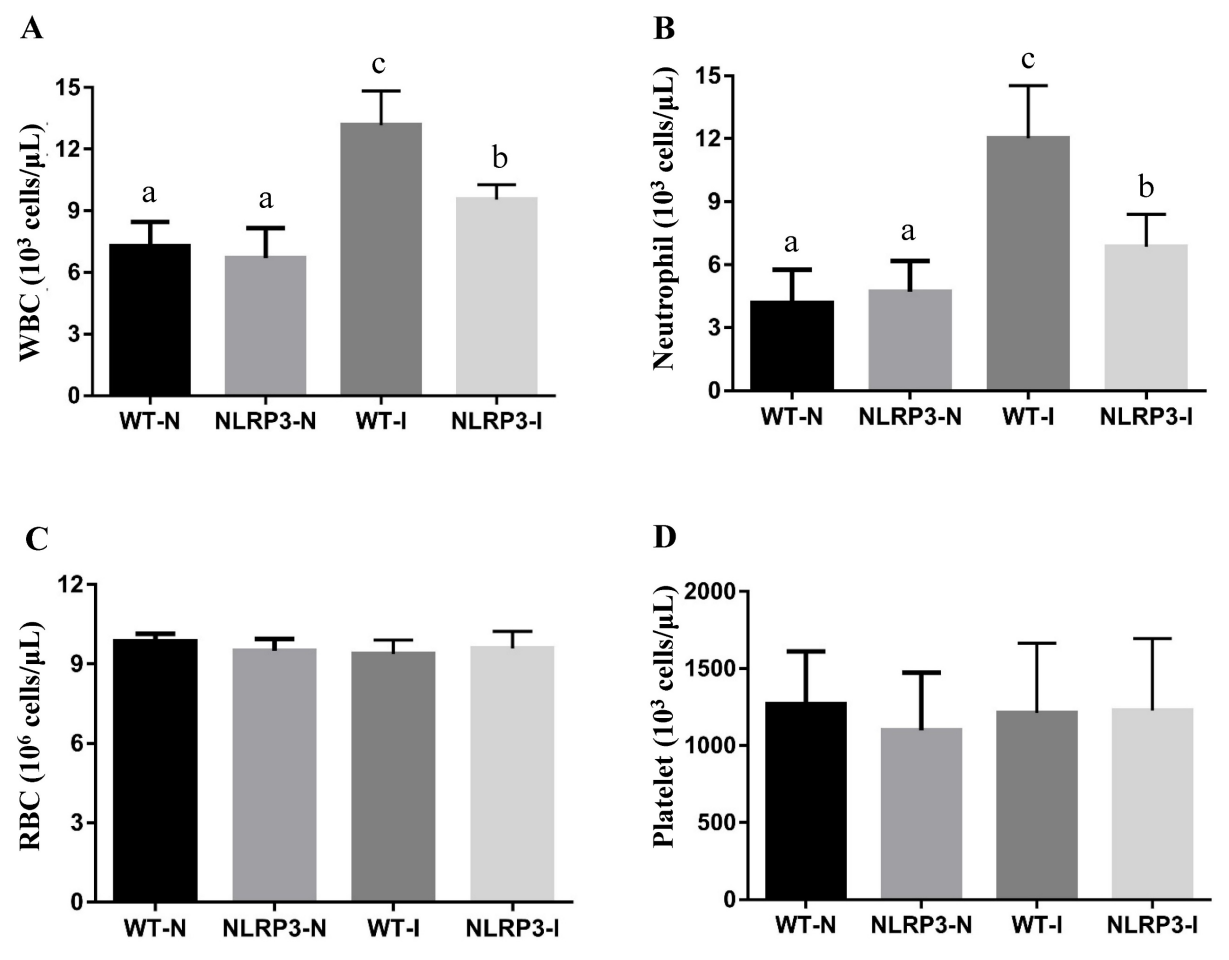
The effects of mass-drop injury (MDI) on complete blood count on *Nlrp3*-knockout and C57BL/6Narl mice. (A) White blood cell (WBC), (B) neutrophil, (C) red blood cell (RBC), and (D) platelet, at 192 h post-contusion. '-I', with injury; '-N', non-injured control. Different lowercase superscript letters identify groups with significant differences (*p* < 0.05).

CBC analysis at 192 h, carried out to assess the hematological impact of MDI, revealed significant differences in white blood cell (WBC) and neutrophil counts among the groups. WBC count was markedly higher in the WT-I group than in the NLRP3-I and non-MDI (WT-N and NLRP3-N) groups; while it was significantly lower in the NLRP3-I group than in the WT-I group, it was notably higher than in the WT-N and NLRP3-N groups (Figure 3A). Similarly, the neutrophil count was significantly elevated in both the WT-I and NLRP3-I groups relative to their respective controls, although it was significantly lower in the NLRP3-I group than in the WT-I group (Figure 3B). No statistically significant differences were found in red blood cell or platelet counts among the groups (Figure 3C, D).

### Histopathology

**Figure 4:**
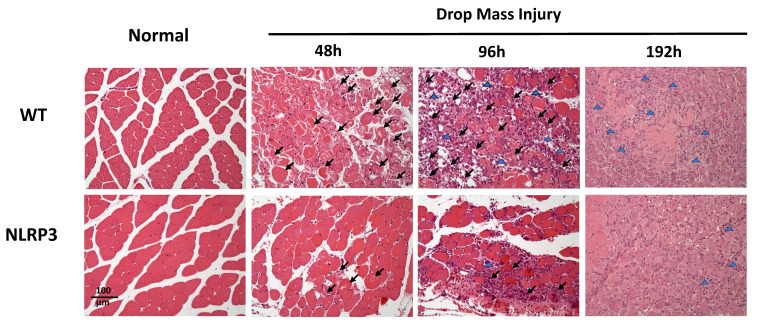
Muscle histopathogenesis following mass-drop injury (MDI) in *Nlrp3-*knockout and C57BL/6Narl wild-type mice. Following MDI, gastrocnemius muscle tissue was collected at 0, 48, 96, and 192 h and transverse sections stained with hematoxylin and eosin. Arrows, large areas of muscle fiber necrosis and degeneration, with swelling, fragmentation, loss of striation, and glassy degeneration. Blue triangles, sparse elongated fibroblasts in the lesion (×200 magnification).

Histological examination of HE-stained tissue samples from the WT-I group at 48 h revealed large areas of muscle fiber necrosis and degeneration, accompanied by swelling, fragmentation, loss of striation, and glassy degeneration (Zenker's necrosis) in the affected regions (arrows, Figure 4). In contrast, the NLRP3-I group exhibited only mild to moderate pathological changes consistent with muscle injury at 48 h. At 96 h, the WT-I group showed extensive infiltration of inflammatory cells (primarily mononuclear macrophages, with a few polymorphonuclear neutrophils indicated by arrows) in the lesion, which also exhibited numerous myoblasts, characterized by scant cytoplasm and hyperchromatic nuclei. In contrast, the NLRP3-I group exhibited less severe inflammatory cell infiltration and fewer myoblasts than the WT-I group. At 192 h, in the WT-I group, most of the necrotic striated muscle fibers had been replaced by newly formed muscle fibers containing nuclei, although a few elongated fibroblasts (indicated by triangles) were present in the lesion; in the NLRP3-I group, in contrast, only new muscle fibers were present, without no evidence of fibroblasts. These findings suggest that muscle injury was less severe in the NLRP3-I group than in the WT-I group. No pathological changes were observed in the muscle tissue of the WT-N or NLRP3-N groups.

### IHC staining of TNF-α, IL-6, CD68, and CD206, and Caspase-3

**Figure 5:**
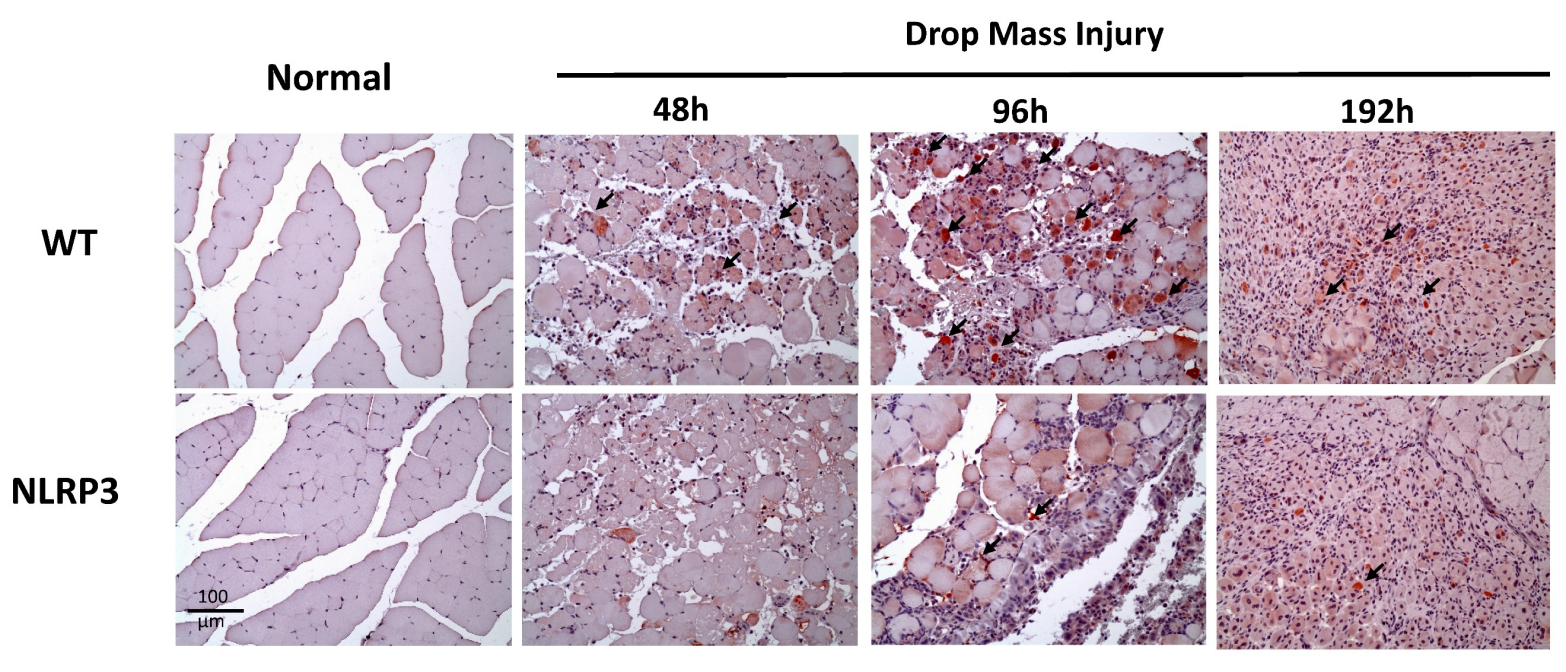
TNF-α expression after mass-drop injury (MDI) in *Nlrp3*-knockout and C57BL/6Narl wild-type (WT) mice. Following MDI, gastrocnemius muscle tissue was collected at 0, 48, 96, and 192 h and transverse sections stained with TNF-α. Arrows, TNF-α-positive cells (×200 magnification).

**Figure 6:**
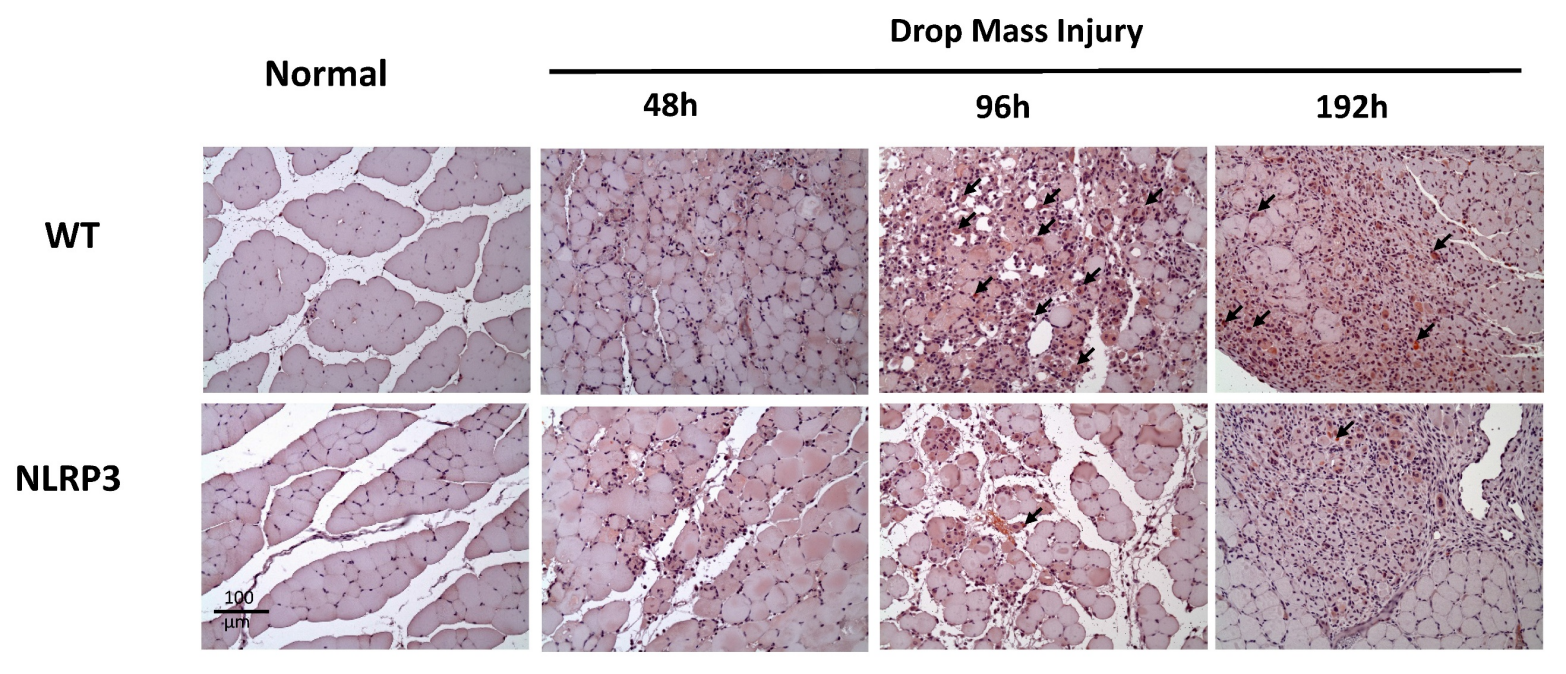
IL-6 expression after mass-drop injury (MDI) in *Nlrp3*-knockout and C57BL/6Narl wild-type (WT) mice. Following MDI, gastrocnemius muscle tissue was collected at 0, 48, 96, and 192 h and transverse sections stained with IL-6. Arrows, IL-6-positive cells (×200 magnification).

**Figure 7:**
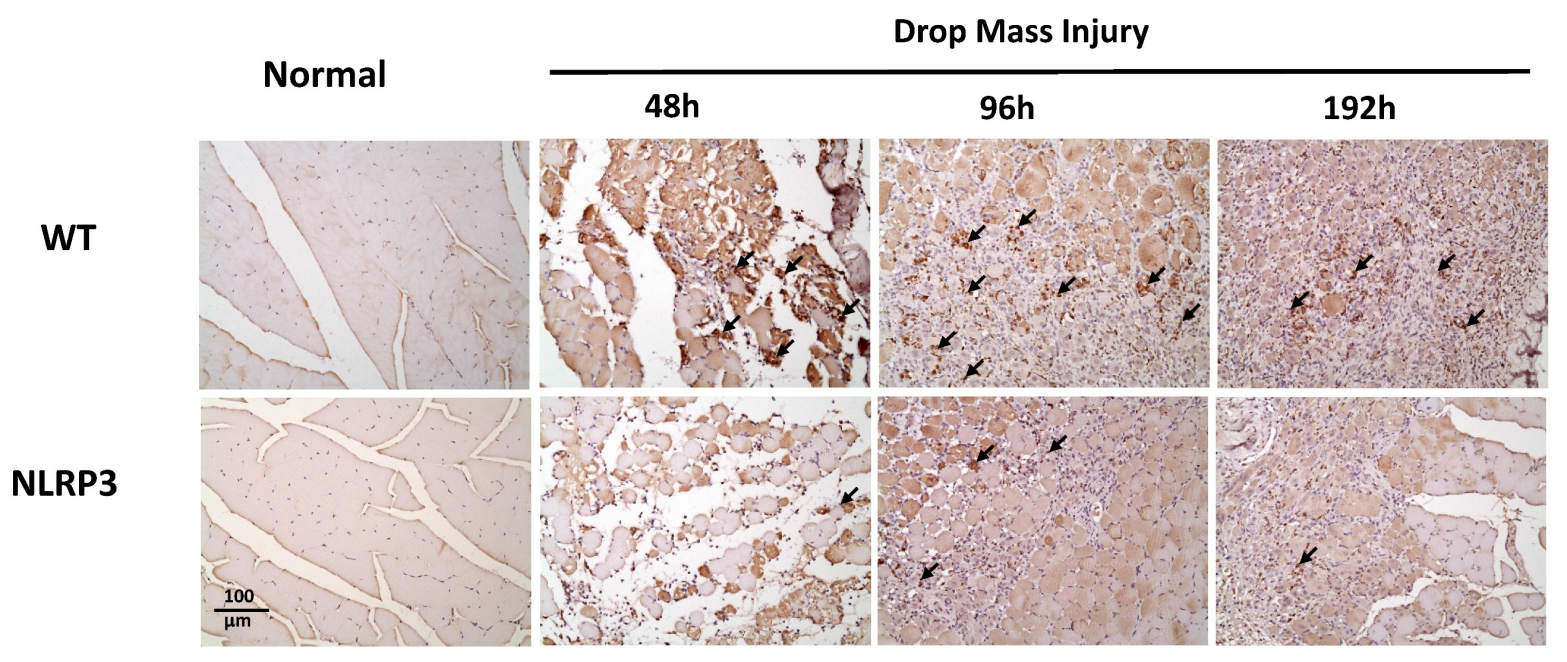
CD68 expression after mass-drop injury (MDI) in *Nlrp3*-knockout and C57BL/6Narl wild-type (WT) mice. Following MDI, gastrocnemius muscle tissue was collected at 0, 48, 96, and 192 h and transverse sections stained with CD68, to reveal the M1 macrophage distribution. Arrows, CD68-positive cells (×200 magnification).

**Figure 8:**
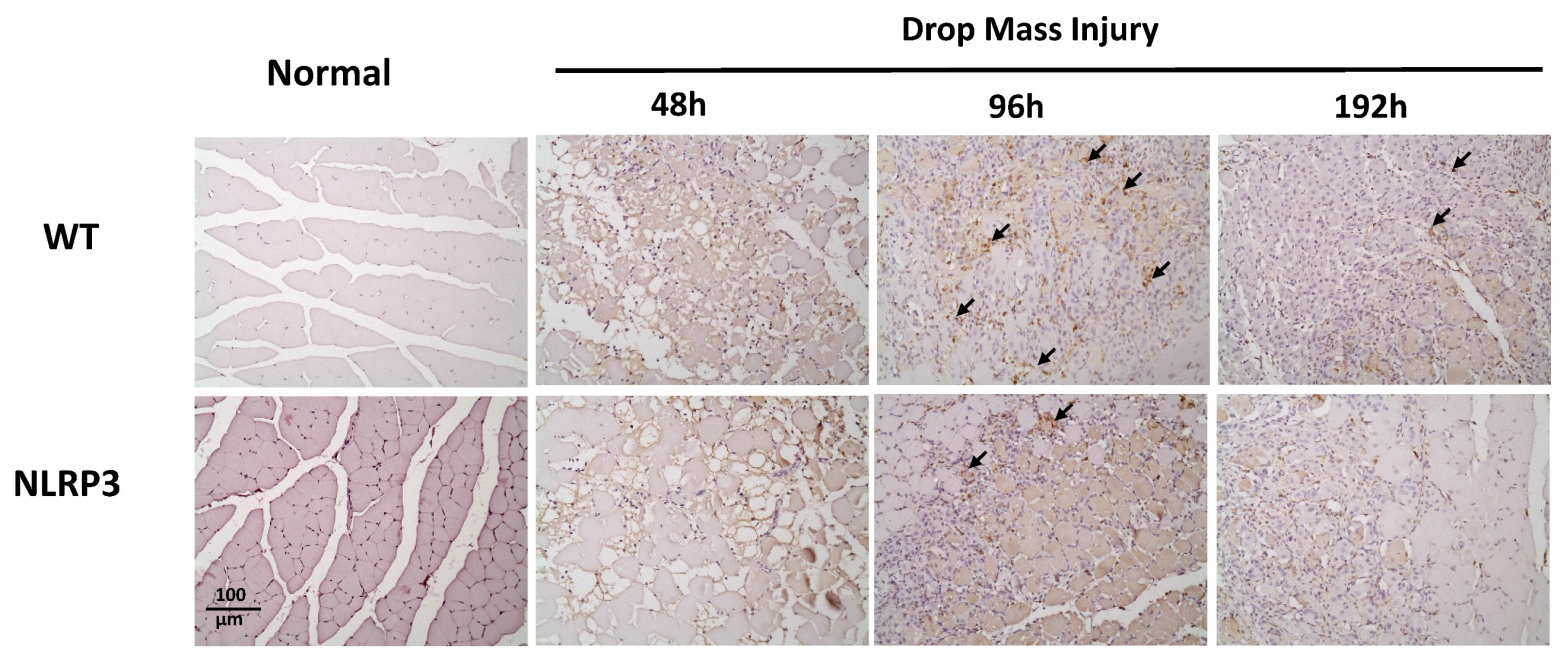
CD206 expression after mass-drop injury (MDI) in *Nlrp3*-knockout and C57BL/6Narl wild-type mice. Following MDI, gastrocnemius muscle tissue was collected at 0, 48, 96, and 192 h and transverse sections stained with CD206, to reveal the M2 macrophage distribution. Arrows, CD206-positive cells (×200 magnification).

**Figure 9:**
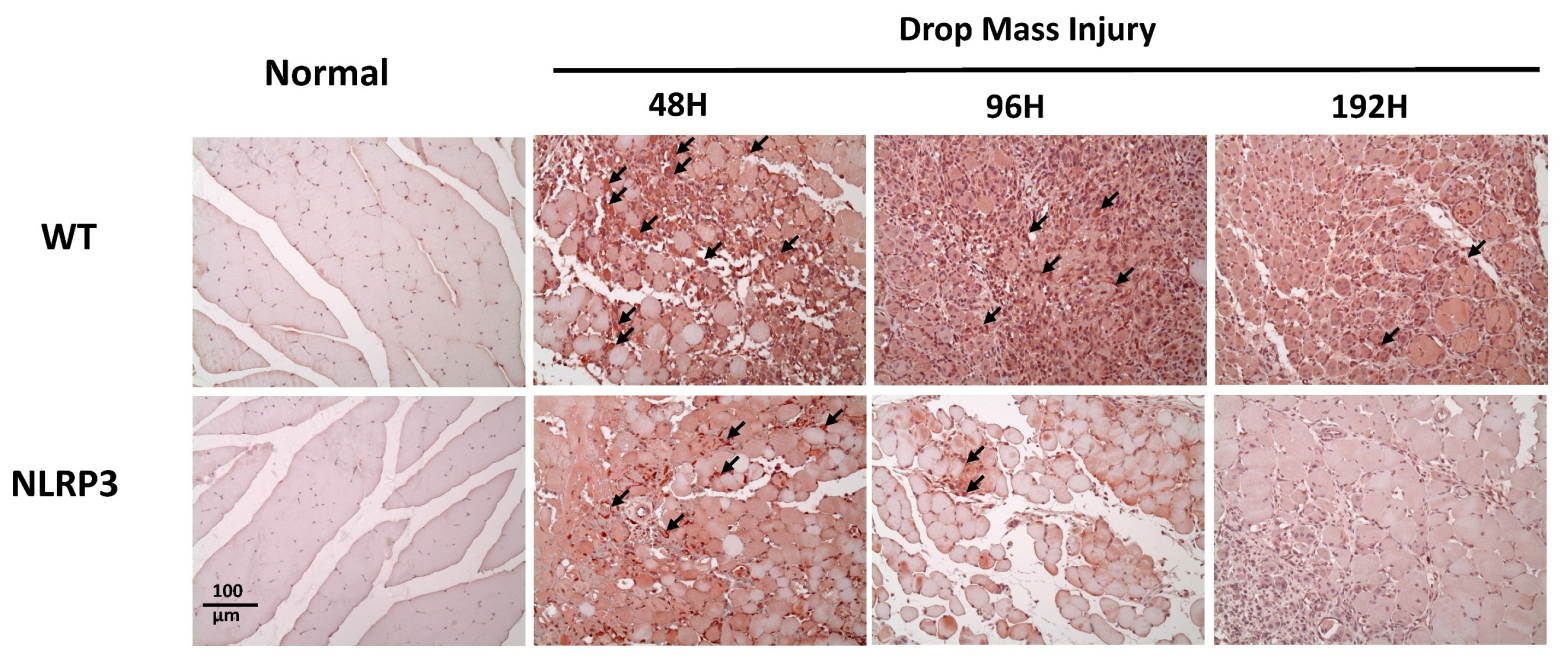
The Caspase-3 (cleavage type) expression after mass-drop injury (MDI) in Nlrp3-knockout and C57BL/6Narl wild-type mice. Following MDI, gastrocnemius muscle tissue was collected at 0, 48, 96, and 192 h and transverse sections stained with Caspase-3. Arrows, Caspase-3 positive cells (×200 magnification).

The injured muscle tissue exhibits positive TNF-α staining (red-brown, indicated by arrows), primarily in the nucleated muscle fibers and certain inflammatory cells. In the WT-I group, TNF-α expression was detectable as early as 48 h post-injury, with a significant increase in TNF-α-positive cells observed at 96 h and persisting through 192 h. In contrast, in the NLRP3-I group, only a small number of TNF-α-positive cells were detected at 96 and 192 h (Figure 5). IL-6 expression was observed primarily in nucleated muscle fibers and some inflammatory cells. In the WT-I group, a significant number of IL-6-positive cells were detected at 96 h, with this expression persisting through 192 h. In contrast, the NLRP3-I group exhibited only a small number of IL-6-positive cells at 96 and 192 h (Figure 6). CD68 is widely utilized as a histochemical and cytochemical inflammatory marker, specifically in relation to the presence and activity of monocytes and macrophages. CD68-positive cells, appearing brown-black and predominantly mononuclear macrophages (indicated by arrows), were detectable in the WT-I group as early as 48 h post-injury. A significant increase in CD68-positive cells was observed at 96 h, with the signal persisting through 192 h. In contrast, the NLRP3-I group exhibited only a small number of CD68-positive cells at the 96 and 192 h, with less pronounced staining than in the WT-I group (Figure 7). Levels of CD206-positive cells were significantly higher at 96 and 192 h in the WT-I group than in the NLRP3-I group (Figure 8). Active Caspase-3 expression exhibited a pronounced elevation in the WT-I group, peaking at 48 hours post-injury and subsequently declining progressively from 96 to 192 hours. A comparable temporal pattern was detected in the NLRP3-I group; however, the magnitude of expression was markedly attenuated relative to the corresponding time points in the WT-I group (Figure 9).

### Masson's trichrome staining for fibrosis

**Figure 10:**
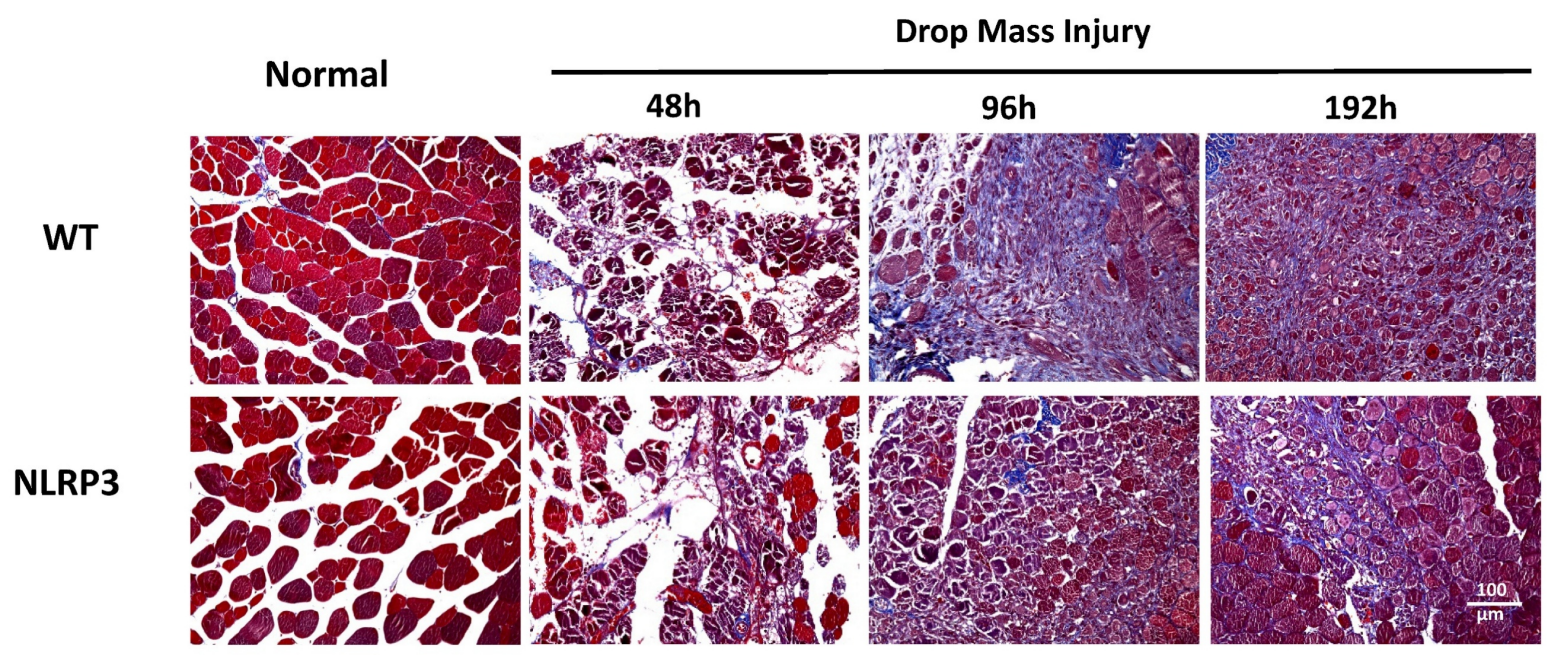
Detection of collagen and fibrosis following mass-drop injury (MDI) in *Nlrp3*-knockout and C57BL/6Narl wild-type mice. Following MDI, gastrocnemius muscle tissue was collected at 0, 48, 96, and 192 h and transverse sections stained with Masson's trichrome stain for differential staining of collagen and muscle fiber. Connective tissue and fibrotic tissue are stained light blue (×200 magnification).

Histopathological evaluation through Masson's trichrome staining demonstrated distinct tissue-specific chromatic differentiation: normal muscle fibers exhibited characteristic reddish-brown coloration, while collagen-rich connective tissue and fibrotic lesions consistently manifested light blue chromatic properties. Blue-stained connective tissue first appeared in the WT-I group at 96 h, with the severity of fibrosis progressively increasing until 192 h. In contrast, NLRP3-I group exhibited only moderate fibrosis at 192 h (Figure 10).

## Discussion

MDI animal models effectively replicate the contusion injuries commonly encountered in sports, reproducing both their physiological responses and histopathological characteristics. The NLRP3 inflammasome plays a pivotal role in modulating inflammatory and immune responses by activating caspase-1, which in turn promotes the maturation and release of proinflammatory cytokines such as IL-1β and IL-18. These mediators participate in both protective immune responses and tissue repair. It has been suggested that cold water immersion after eccentric muscle contraction may regulate NLRP3 activation by upregulating ubiquitin-proteasome system-related proteins 19. Nonetheless, the role of NLRP3 in structural muscle damage during physical injury remains unclear. In the present contusion model, *Nlrp3*-KO individuals exhibited reduced muscle injury and inflammation, based on histopathological and physiological assessments. NLRP3 plays a key role not only in traumatic brain and spinal cord injury but also in sports-related musculoskeletal injury.

Skeletal muscle injuries are extremely common, accounting for approximately 10%-35% of sports injuries 20. In a prior study, relative to non-injured controls, MDI-induced contusion models exhibited significantly elevated serum CK and LDH at 192 h post-contusion, whereas AST levels were not significantly affected 17. In the present study, CK and LDH levels remained significantly elevated from 48 to 192 h post-injury; AST levels, in contrast, were significantly elevated at 48 and 96 h, but returned to baseline by 192 h. In muscle injury resulting from completing a full marathon, serum CK and AST peaked on day 1 after the race, while serum LDH peaked immediately post-race 21. Levels of muscle injury-related markers change over time not only after intensive exercise but also after substantive muscle damage. For instance, in an isoprenaline-induced cardiac infarction model, tiron, an antioxidant and iron chelator, significantly reduced serum CK-MB, LDH, and AST and downregulated immune responses involving IL-1β, NF-κB, TLR4, and iNOS in infarcted cardiac tissues; it also effectively mitigated NLRP3 inflammasome activation and inhibited the TLR4/NF-κB/iNOS signaling cascade, thereby alleviating oxidative and inflammatory stress 22. As muscle serum markers, LDH, CK, myoglobin, and AST serve as valuable indicators for monitoring metabolic adaptations to physical exercise; such monitoring provides critical insights into muscular workload and the potential for exercise-induced muscle damage 23. Here, at 96 and 192 h post-contusion, the NLRP3-I group exhibited significantly lower serum CK levels than the WT-I group, while its LDH levels were lower only at 192 h, suggesting that the myofibril damage is attenuated through *Nlrp3* knockout.

The NLRP3 inflammasome regulated the maturation and secretion of the cytokine IL-1β. This signaling pathway functions by binding IL-1β to the IL-1 receptor, thereby activating the IL-1 receptor-associated kinase 1 and subsequently the transcription factor NF-κB. A study of sepsis-induced muscle atrophy in a mouse model revealed that the NLRP3 inflammasome participates in regulating muscle atrophy, as NLRP3-KO mice exhibited less severe muscle atrophy and lower serum IL-1β levels than WT mice. Based on those findings, inhibiting the NLRP3 inflammasome and its activation of the IL-1β pathway may help prevent inflammation-induced muscle atrophy in septic patients 24. Klotho, which mitigates diabetes-induced elevation of serum creatine kinase-muscle/brain (CK-MB) and LDH, cardiac fibrosis, cardiomyocyte apoptosis, and cardiac dysfunction, also inhibits NLRP3 activation and TNF-α, IL-1β, and IL-18 expression. Its protective effects in diabetes-induced cardiac injury are therefore associated with suppression of the NLRP3 inflammasome pathway 25. In a study comparing inflammatory cytokine expression 24, 72, 168, and 336 h after muscle contusion by heavy object impact, analysis of qPCR data revealed significantly elevated TNF-α expression in the contusion group at 24 and 72 h post-injury, persisting through 168 and 336 h, relative to the non-injured controls 26. This is consistent with our finding of elevated TNF-α expression in the wild-type mice after impact injury.

The role of macrophages in skeletal muscle regeneration has been demonstrated 8, 27. While M1 macrophages are associated with muscle necrosis, M2 macrophages are associated with regenerative myofibers and participate in inhibiting fibro-adipogenic progenitors and promoting muscle regeneration. Here, we examined changes in the levels of CD68, an M1 marker, after muscle contusion in *Nlrp3*-KO and WT mice. In a previous study, the phytocompound berberine significantly downregulated the expression of the NLRP3 inflammasome and its associated molecules in macrophages, thereby attenuating NLRP3 inflammasome activation-induced M1 macrophage polarization and subsequent inflammation. The neuroprotective effects of berberine were closely associated with suppression of inflammation following peripheral nerve injury; berberine achieved these effects by inhibiting NLRP3 activation-induced M1 macrophage polarization 28. Imbalance in macrophage phenotypes and dysregulation of the M1-M2 transition contribute substantially to the persistence of inflammation and impaired tissue regeneration. Biomaterials and biomaterial-based delivery systems offer promising strategies to regulate macrophage polarization 29. Notably, M2 macrophages play a pivotal role in facilitating tendon tissue regeneration and remodeling by promoting cellular proliferation, adipocyte differentiation, and extracellular matrix component regulation 30. In the present study, M1 macrophages responded first to contusion, reaching the injury site 48-96 h post-injury in the WT-I group. In *Nlrp3*-KO mice, the number of M1 macrophages decreased in the early inflammatory phase after skeletal muscle injury. At 96 and 192 h post-injury, the WT-I group exhibited significantly more CD206-positive cells than the NLRP3-I group. These findings suggest a mechanism whereby inflammatory balance contributes to tissue adaptation and repair following injury induction.

The sequence of immune cell invasion after muscle contusion is similar to that observed in other injury models; it involves expression of inflammatory cytokines such as TNF-α and IL-6, which can activate macrophages and mediate inflammatory responses 31. Both damaged myocytes and activated macrophages exhibit substantial expression of IL-6 and TNF-α in the early stages of muscle injury, suggesting that M1 macrophages might be related to the cytokines secreted after injury 32. Here, IL-6-positive cells were observed in nucleated muscle fibers and inflammatory cells in lesions. In the WT-I group, many IL-6-positive cells were observed from 96 h after injury, persisting until 192 h. In contrast, in the NLRP3-I group, few IL-6 positive cells were present at 96 h and 192 h. These results are consistent with previous finding that inhibiting IL-6 expression reduces the inflammatory response of muscle tissue and fibrosis severity. Signaling pathways downstream of IL-6 (the IL-6/Stat3 and IL-1β/Egr1 pathways) are closely associated with fibrosis 33. In synthesis, we propose that downregulation of *Nlrp3* expression might reduce muscle fibrosis by modulating the IL-6 fibrosis-related pathway.

Exercise interventions can regulate the NLRP3 inflammasome. In osteoarthritis, moderate-intensity exercise reduces inflammation and pyroptosis by increasing the expression of Metrnl, an adipomyokine that inhibits the PI3K/Akt/NF-κB signaling pathway and subsequently the NLRP3/caspase-1/GSDMD pathway 34. Exercise training is widely recognized as a physiological stressor that elicits beneficial cellular adaptations, ultimately inducing a cardioprotective phenotype. Accordingly, regular exercise confers protection against myocardial injury. The mechanisms underlying exercise-induced cardiac preconditioning involve activation of the anti-radical defense system, enhanced nitric oxide production, and the modulation of opioids, myokines, and adenosine-5'-triphosphate (ATP)-dependent potassium channels 35. Notably, in myocardial ischemia-reperfusion injury, regular exercise has been shown to downregulate NLRP3 expression in cardiac tissue, thereby mitigating NLRP3-mediated pyroptosis and contributing to myocardial protection 36. Aerobic exercise alleviates pyroptosis in endothelial cells, adipocytes, and hippocampal cells, thereby mitigating pyroptosis-related diseases by inhibiting NLRP3 37. In an animal model of ventilator-induced lung injury (VILI), PKC activation increased NLRP3 levels, leading to lung injury; exercise was found to reduce lung injury by inhibiting PKC and NLRP3 activation, suggesting it as a potential clinical strategy for preventing VILI 38. Exercise interventions thus yield positive physiological benefits by downregulating NLRP3. Consistent with this, we found that *Nlrp3* knockdown reduced inflammation and accelerated recovery following MDI-induced muscle injury. Nonetheless, the downregulation of NLRP3 should be further investigated in relation to exercise performance under conditions of injury. At 192 h, the NLRP3-I group exhibited significantly less fibrotic tissue, based on Masson's trichrome staining, than the WT-I group (Figure 10), indicating that NLRP3 knockout might improve fibrotic gene expression in mice with contusion-induced muscle injury. We therefore hypothesize that reduced NLRP3 expression might improve muscle recovery by increasing the expression of myogenic genes such as *Myod* and *Pax-7* in muscle cells and reducing fibrogenic gene expression during muscle repair after contusion-induced muscle injury 39. The molecular basis of the function of NLRP3 thus requires further investigation, potentially using an *Nrlp3*-knockout animal model of muscular injury.

Mouse models of MDI-induced muscle contusion provide a valuable tool for investigating the mechanisms of muscle recovery and evaluating the efficacy of therapeutic interventions. Here, in *Nrlp3*-KO mice, the inflammatory response was inhibited, reducing contusion-induced muscle damage and fibrosis. This mechanism may involve regulation of downstream cytokines such as TNF-α and IL-6, leading to reduced inflammation and increased muscle regeneration. *Nlrp3* thus provides a potential target for pharmaceutical development to improve treatment outcomes and recovery in muscle injury in sports. Novel screening techniques and functional assays are required to test the new anti-NLRP3 compounds being developed in the rapidly expanding field of inflammasome biology in musculoskeletal health 40. Moreover, the potential for developing ergogenic aids that alter *Nlrp3* regulation warrants further investigation. However, the possibility that such drugs or supplements would be prohibited by the World Anti-Doping Agency requires consideration.
